# Digital Technology Characteristics and Literacy Among Families With Children With Asthma: Cross-Sectional Study

**DOI:** 10.2196/48822

**Published:** 2023-11-29

**Authors:** Kristin Kan, Lu Morales, Avani Shah, Emily Simmons, Leonardo Barrera, Liana Massey, Greta List, Ruchi S Gupta

**Affiliations:** 1 Division of Advanced General Pediatrics and Primary Care, Feinberg School of Medicine, Northwestern University, Chicago, IL, United States; 2 Center for Food Allergy and Asthma Research, Institute of Public Health and Medicine, Feinberg School of Medicine, Northwestern University, Chicago, IL, United States; 3 Mary Ann & J Milburn Smith Child Health Outcomes, Research, and Evaluation Center, Ann & Robert H Lurie Children’s Hospital of Chicago, Chicago, IL, United States; 4 Division of Pulmonary and Sleep Medicine, Feinberg School of Medicine, Ann & Robert H Lurie Children’s Hospital of Chicago, Chicago, IL, United States; 5 Brown University, Providence, RI, United States

**Keywords:** pediatric asthma, digital literacy, health equity, equity, asthma, respiration, respiratory, pulmonary, child, children, youth, survey, surveys, disparity, disparities, socio-demographic, sociodemographic, use, technology use, self-management, family

## Abstract

**Background:**

The use of digital technology in pediatric asthma management has emerged as a potential tool for improving asthma management. However, the use of digital tools has the potential to contribute to the inequitable delivery of asthma care because of existing social factors associated with asthma disparities. Our study focused on parents’ chosen language and sociodemographic factors that might shape the use of digital technology in asthma self-management.

**Objective:**

This study aims to estimate and compare patient, family, and technology-related characteristics by parents’ chosen language (English or Spanish) and compare a digital literacy measure by sociodemographic factors.

**Methods:**

Survey data were collected from July to December 2021 from parents of children with asthma who were seen by a Chicago pediatric health system pulmonary provider. Questions assessed patient and family characteristics, digital technology use, and digital literacy, measured using the validated eHealth Literacy Scale (eHEALS). Chi-square tests and multivariable logistic regression were used for comparisons, and Kruskal-Wallis tests were used for comparing median eHEALS scores by social characteristics.

**Results:**

Of the 197 parents surveyed, 24.4% (n=49) of parents identified as a race categorized as other, 37.1% (n=67) as White, and 38.6% (n=75) as Black; 47.2% (n=93) identified as Hispanic/Latino/Latina. Additionally, 79.7% (n=157) of parents preferred English, and 20.3% (n=40) preferred Spanish. English-speaking parents were more likely to report having a data plan for their smartphone (117/157, 74.5%) or high-speed internet (138/157, 87.9%) compared to Spanish-speaking parents (smartphone: 23/40, 58%; *P*=.03; internet: 27/40, 68%; *P*=.002). Compared with Spanish-speaking parents, English-speaking parents were less likely to report having a lot or some concern about paying for internet (28/40, 70% vs 83/157, 52.9%; *P*=.046) or about data privacy (35/40, 88% vs 105/157, 67.5%; *P*=.01). Digital literacy scores differed significantly by race, income, education level, and language. In a multivariable model, language was not a significant factor for having high-speed internet service (*P*=.12) or concern about paying for internet at home (*P*=.60), but it was a significant factor for concerns about data privacy (*P*=.04).

**Conclusions:**

The significant differences in technology-related characteristics suggest that digital connectivity, affordability, and data privacy may also be important factors in considering digital technology use in asthma care.

## Introduction

Digital technology is emerging as a tool to help manage pediatric chronic disease. Studies have shown that disease self-management is improved by the use of smartphone apps and remote monitoring devices, as evidenced by increased medication adherence, improved attendance at medical appointments, and improved measures of quality of life [1,2].

There are similarly promising findings for pediatric asthma management with digital technologies; however, the availability and use of digital technology in asthma care could be inequitable [3-5]. Asthma disparities due to sociodemographic factors, including low socioeconomic status, race, ethnicity, and household language, are well-described [6-9]. Implementing digital technology in the context of existing disparities could potentially widen disparities already experienced in asthma care. For example, previous studies of general digital technology use have found that Black patients reported using mobile technology for social activities, but fewer used it for health-related information or communication [10]. Furthermore, a study of a large urban area found disparities in digital connectivity for Hispanic populations [11]. Thus while digital health care may have advantages for tailoring health information and education, an intentional design to meet the linguistic, cultural, and literacy needs of specific populations is necessary [12]. Implementation without attention to these known social determinants of health, associated with disparate asthma care, could lead to unintentional perpetuation, or even worsening, of disparities.

In partnership with pediatric pulmonary providers that serve a primarily low-income and racial-ethnically diverse population, our study team examined key characteristics that might influence families’ digital technology use for asthma care. In particular, we highlighted the differences in technology-related characteristics between English-speaking and Spanish-speaking families with children with asthma. Language-concordant care is an essential component of high-quality health care in the United States [13,14]. Studies have typically focused on the use of medical interpreters in care delivery, and only a few studies have compared the use of digital health technology among patients with a non-English preference [15]. They found that Spanish speakers tend to have lower digital literacy than English speakers and that there are cultural differences in what they want from health tools [16,17].

Health systems and clinicians need to understand how to support the equitable delivery of digital health for families with children with asthma as digital engagement expands in health care delivery [18]. To inform those efforts, we surveyed parents/caregivers about their digital technology access, use, and preferences at home and in their community. Our study focuses on parent language and other sociodemographic factors that might shape the use of digital technology in asthma self-management.

## Methods

### Study Procedures and Participants

For this cross-sectional study, data collection occurred from July 2021 to December 2021. A convenience sample of parents/caregivers (henceforth, parents) of patients with an asthma diagnosis, managed by pediatric pulmonology providers at a single pediatric hospital system, were recruited by email and in person at a clinic. If the child (ie, patient) was seen by a pediatric pulmonology provider during the study period, then their parent was invited to fill out the survey by email. For patients approached at the pediatric asthma clinic, a research staff member asked parents while they were waiting to be seen by their pulmonary provider if they were interested in completing the survey. If they agreed, then the unique survey link was opened on a tablet for completion. The pediatric asthma clinic was located in Chicago, Illinois, whereas other pulmonary clinics, which manage all pulmonary conditions, were located in Chicago and the surrounding suburbs. The characteristics of participants who completed the web-based survey versus the in-person survey are included in Table S1 in Multimedia Appendix 1. We recruited by email and in person to ensure that our sample was not biased toward only those who felt comfortable participating by email. In our previous research, patients expressed that meeting research staff in person was an important component of study participation. While the overall response rate was 47.2% (197/417 participants approached), the response rate was higher in a clinic (81/100, 81% of participants approached) than by email (116/317, 36.6% of participants emailed). During the study period, approximately 1000 unique pediatric patients with asthma were managed by pulmonary providers in our health system, so our sample size represented 20% of that patient population.

To be eligible, the parents had to complete the survey in English or Spanish and have a child with an asthma diagnosis who was younger than 18 years. Parents were excluded if their child had a comorbidity, making the management of asthma different from typical asthma management per the pulmonology provider’s clinical judgment (eg, ventilator dependent or interstitial lung disease).

Asthma diagnosis was retrieved from the pulmonology visit’s associated *International Classification of Diseases, Tenth Revision* (*ICD-10*) code in the patient’s chart from the electronic health record. Participants were compensated with a US $10 electronic gift card. Language preference for survey participation was indicated by selecting English or Spanish when asked “What is your preferred language of communication?” [15].

### Ethical Considerations

The Lurie Children’s Hospital of Chicago Institutional Review Board deemed the study exempt from review (2021-4330).

### Instrument

The survey was developed with pediatric primary care and pulmonology expertise. Questions evaluated patient and family characteristics [19,20] (parent and child gender, race, and Hispanic/Latino/Latina ethnicity; household income; child grade; and perceived burden from asthma) and digital technology access and use (devices [21], activities on devices, type of internet access [21], concern paying for internet, concern about data privacy, and interest in technology for asthma management; the survey is available in Multimedia Appendix 2 [19-21]). Questions were pretested with research staff, not affiliated with the study, and parents to ensure clarity and reliability regarding the function of the questions before dissemination.

Additionally, digital literacy was measured in the survey using the validated eHealth Literacy Scale (eHEALS), which was developed from social cognitive theory and self-efficacy theory, and is the most commonly used assessment of an individual’s ability to use digital resources for health [22-26]. The eHEALS is composed of 8 items measured using a 5-point Likert scale, varying from “strongly agree” (5 points), “agree” (4 points), “neutral” (3 points), “disagree” (2 points), and “strongly disagree” (1 point). The total eHEALS score for a participant completing all items ranged from 8 to 40 by a summation of every item’s score, and a higher score meant better digital literacy. The English- and Spanish-validated versions of eHEALS were used.

### Statistical Analysis

Descriptive statistics of key sociodemographic factors and survey responses included frequencies and proportions. Based on distribution for self-identified race, there was further categorization into 3 major groups: Black or African American, White, or other (Asian, American Indian/Alaskan Native, Hawaiian/Pacific Islander, or preferred to self-describe). The child’s race was categorized similarly. The estimated household income variable was dichotomized (ie, <US $50,000 or ≥US $50,000) at approximately 200% of the federal poverty level of an annual 2022 income for a household of 4 persons (US $55,500) in the United States [27]. Parent education was dichotomized into two categories: high school education or less and any college education or more, including graduate-level education. Child grade was dichotomized from early child education (kindergarten) to the end of middle school (eighth grade) and high school (ninth to 12th grade). Asthma diagnosis was determined by the *ICD-10* code associated with the pulmonary clinic visit and categorized into mild, moderate, or severe based on the visit’s coding.

Chi-square tests were used for bivariate comparisons for categorical variables unless there was a small response size, then Fischer exact tests were used. A multivariable logistic regression with parent language (ie, English as the referent group vs Spanish) and dichotomized household income, as defined above, was used to look at three dichotomized dependent variables: has high-speed internet (0 was no and 1 was yes), concern about paying for internet at home during the COVID-19 pandemic (0 for “Not too much/at all/do not have to pay” and 1 for “A lot/Some”), and concern about data privacy (0 for “Not too much/at all/do not have to pay” and 1 for “A lot/Some”). Kruskal-Wallis tests were used for comparing median eHEALS scores by categorical variables. Nonparametric statistical tests were used for the analysis given the small sample size. Results are reported as significant for 2-sided *P* values <.05. Analyses were performed using SPSS, Version 28 (IBM Corp).

## Results

### Characteristics of Parent and Child

Of the 197 parent-child dyads surveyed, 89.1% (n=172) were female parents, and 10.4% (n=20) were male parents (Table 1). The median age was 37 (IQR 32-43) years. Surveyed parents identified their race as other (n=49, 24.4%), White (n=67, 37.1%), or Black or African American (n=75, 38.6%), and 47.2% (n=93) identified their ethnicity as Hispanic/Latino/Latina. By language, there were statistical differences in parent education level and income. While 68.8% (108/157) of English-speaking parents reported having at least some college education, only 18% (7/40) of Spanish-speaking parents reported similar education levels (*P*<.001). Further, 33.1% (48/157) of parents who preferred English and 9% (3/40) of parents who preferred Spanish reported estimated household incomes greater than US $50,000 (*P*=.004).

**Table 1.** Characteristics of parent and child by language (N=197).

		Overall sample (N=197)	English (n=157)	Spanish (n=40)	*P* value
Parent age (years; n=181), median (IQR)		37 (32-43)	35 (31-42)	41 (37-45)	.04
**Parent gender**^**a**^ **(n=193), n (%)**					.26
	Male	20 (10.4)	14 (9.2)	6 (15.4)	
	Female	172 (89.1)	139 (90.8)	33 (84.6)	
**Parent race (n=191), n (%)**					<.001
	Black or African American	75 (38.6)	75 (49.0)	0 (0.0)	
	White	67 (37.1)	52 (34.0)	15 (39.5)	
	Other (Asian, American Indian/Alaskan Native, Hawaiian/Pacific Islander)	49 (24.4)	26 (17.0)	23 (60.5)	
Parent ethnicity, Hispanic/Latino/Latina (yes), n (%)		93 (47.2)	54 (35.3)	39 (97.5)	<.001
**Parent education, n (%)**					<.001
	High school or less	82 (41.6)	49 (31.2)	33 (82.5)	
	Any college or more	115 (58.4)	108 (68.8)	7 (17.5)	
**Estimated annual household income (US $; n=180), n (%)**					.004
	<50,000	129 (65.5)	97 (66.9)	32 (91.4)	
	≥50,000	51 (25.9)	48 (33.1)	3 (8.5)	
**Child gender, n (%)**					.12
	Male	124 (62.9)	103 (65.6)	21 (52.5)	
	Female	73 (37.1)	54 (34.4)	19 (47.5)	
**Child race (n=191), n (%)**					<.001
	Black or African American	79 (40.1)	79 (52.0)	0 (0.0)	
	White	75 (38.1)	51 (33.6)	24 (61.5)	
	Other (Asian, American Indian/Alaskan Native, Hawaiian/Pacific Islander)	37 (18.8)	22 (14.5)	15 (38.5)	
Child ethnicity, Hispanic/Latino/Latina (yes), n (%)		92 (46.7)	54 (34.8)	38 (97.4)	<.001
**Child grade (2021-2022; n=196), n (%)**					.78
	Kindergarten to 8th grade	174 (88.8)	138 (88.5)	36 (90.0)	
	9th to 12th grade	22 (11.2)	18 (11.5)	4 (10.0)	
**Difficulties caused by asthma, n (%)**					.02
	Minor	63 (32.0)	43 (27.4)	20 (50.0)	
	Moderate	73 (37.1)	60 (38.2)	13 (32.5)	
	Severe	61 (31.0)	54 (34.4)	7 (17.5)	
**Asthma diagnosis (n=196), n (%)**					.77
	Mild	27 (13.8)	22 (14.1)	5 (12.5)	
	Moderate	51 (26.0)	42 (26.9)	9 (22.5)	
	Severe	118 (60.2)	92 (59.0)	26 (65.0)	

a One participant selected “other/preferred not to answer.”

Among the 197 children, 88.8% (n=174) of them were between kindergarten and eighth grade, and 11.2% (n=22) were between ninth and 12th grade (Table 1). When evaluating the self-reported burden experienced by the family from their child’s asthma, 32% (n=63) reported minor, 37.1% (n=73) reported moderate, and 31% (n=61) reported severe difficulties. The asthma severity diagnosis distribution, however, was 13.8% (n=21) mild, 25.9% (n=51) moderate, and 60.2% (n=118) severe. While there were statistical differences in the perceived difficulties caused by asthma by language participation (42/157, 27.4% of English-speaking parents and 50% (20/40) of Spanish-speaking parents reported minor difficulties; *P*=.02), there were no differences in asthma severity diagnosis by language (*P*=.77).

### Technology-Related Characteristics

Most of the 197 parents reported having a smartphone (n=181, 91.9%), with 68.5% (n=135) reporting having a desktop or laptop and 63.5% (n=125) reporting having a tablet computer at home (Table 2). Parents mostly reported that the devices were used for entertainment by their child (n=170, 86.3%); 71.1% (n=140) of parents reported having a cell phone data plan, and 83.8% (n=165) reported having high-speed internet service. More English-speaking parents, compared to Spanish-speaking parents, reported having a cell phone data plan (117/157, 74.5% vs 23/40, 58%; *P*=.03) or having high-speed internet service (138/157, 87.9% vs 27/40, 68%; *P*=.002).

**Table 2.** Technology-related characteristics by language (N=197).

		Overall sample (N=197), n (%)	English (n=157), n (%)	Spanish (n=40), n (%)	*P* value
**Devices at home (yes)^a^**					
	Desktop or laptop	135 (68.5)	110 (70.1)	25 (62.5)	.85
	Smartphone	181 (91.9)	146 (93.0)	35 (87.5)	.26
	Tablet or other portable wireless computer	125 (63.5)	104 (66.2)	21 (52.5)	.11
**Child activities on devices (yes)^a^**					
	Remote learning	122 (61.9)	97 (61.8)	25 (62.5)	.93
	Entertainment (eg, YouTube, games)	170 (86.3)	136 (86.6)	34 (85.0)	.79
	Communication with family and friends	121 (61.4)	98 (62.4)	23 (57.5)	.57
**Types of internet access at home (yes)^a^**					
	Cell phone data plan for a smartphone or other mobile device	140 (71.1)	117 (74.5)	23 (57.5)	.03
	High-speed internet service (eg, cable, fiber optic, DSL^b^ service)	165 (83.8)	138 (87.9)	27 (67.5)	.002
	Satellite internet service^c^	16 (8.1)	9 (5.7)	7 (17.5)	.02
	Some other service^c^	5 (2.5)	2 (1.3)	3 (7.5)	.06
**Concern about paying for internet at home during the COVID-19 pandemic**					.046
	A lot/some	111 (56.3)	83 (52.9)	28 (70.0)	
	Not too much/not at all	77 (39.1)	68 (43.3)	9 (22.5)	
	Do not have to pay for internet	9 (4.6)	6 (3.8)	3 (7.5)	
**Concern about data privacy**					.01
	A lot/some	141 (71.6)	106 (67.5)	35 (87.5)	
	Not too much/not at all	56 (28.4)	51 (32.5)	5 (12.5)	
**Interest in technology for managing your child’s asthma (n=196)**					.29
	A lot/some	154 (78.6)	125 (80.1)	29 (72.5)	
	Not too much/not at all	42 (21.4)	31 (19.9)	11 (27.5)	

a These questions asked participants to select all that apply.

b DSL: digital subscriber line.

c Fisher exact test was used for comparing English- and Spanish-speaking participants due to the small sample responses.

English-speaking parents (68/157, 43.3%) were less likely to have concerns about paying for internet and cell phone service during the pandemic than Spanish-speaking parents (9/40, 23%; *P*=.02). English-speaking parents (106/157, 67.5%) were also less likely to have concerns about data privacy than Spanish-speaking parents (35/40, 88%; *P*=.01). There were no statistically significant differences by language in interest in technology use in asthma care (*P*=.29). In multivariable regression, the associations between having high-speed internet service (adjusted odds ratio [aOR] 0.5, 95% CI 0.2-1.2; *P*=.12) and concern about paying for internet at home (aOR 1.2, 95% CI 0.5-2.8; *P*=.60) with parent language were not significant after adjusting for household income (Table 3). Concerns about data privacy by language remained statistically significant after adjusting for household income (aOR 3.2, 95% CI 1.0-9.7; *P*=.04).

**Table 3.** Associations between technology-related perceptions and language (adjusted for income status).

	Spanish, adjusted odds ratio (95% CI)	*P* value
Has high-speed internet service (eg, cable, fiber optic, or DSL^a^ service)	0.5 (0.2-1.2)	.12
Concern about paying for internet at home during COVID-19 pandemic^b^	1.2 (0.5-2.8)	.60
Concern about data privacy	3.2 (1.0-9.7)	.04

a DSL: digital subscriber line.

b The answers were coded as 0 for “not too much/at all/do not have to pay” and 1 for “a lot/some.” The referent group for language was English.

### Digital Literacy

**Figure 1.**
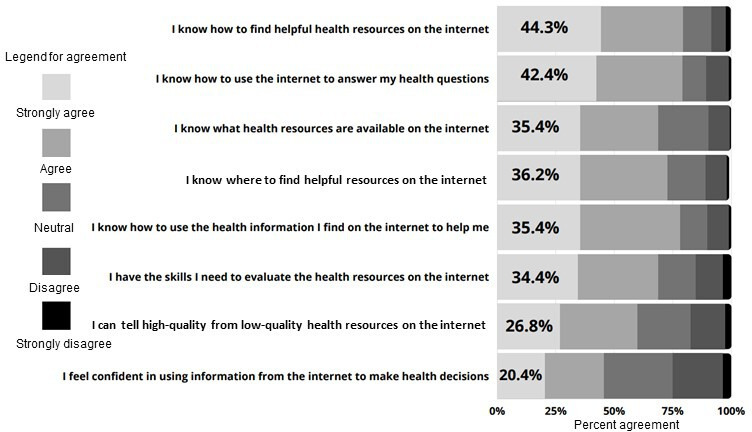
Participant responses to digital health literacy items (N=197).

In an assessment of digital literacy, many of the parents knew how to find helpful health resources on the internet (86/194, 44.3%) and used the internet to answer their health questions (83/196, 42.4%), but only 26.8% (52/194) felt that they could identify high-quality resources from low-quality ones on the internet, and 20.4% (40/196) of respondents felt confident using the information to make health decisions (Figure 1). When examining median eHealth scores, they were significantly different by parent race (*P*<.001), income (categorized as those above and below an estimated annual household income of US $50,000; *P*<.001), education level (*P*<.001), and parent language (*P*<.001; Table 4).

**Table 4.** Association between digital literacy and race, ethnicity, income, education level, and language.

		eHealth score, median (IQR)	*P* value
Overall sample		32 (26-37)	N/A^a^
**Parent race**			<.001
	Black	32 (27-38)	
	White	33 (29-39)	
	Other (Asian, American Indian/Alaskan Native, Hawaiian/Pacific Islander)	30 (24-33)	
**Parent ethnicity Hispanic/Latino/Latina**			.09
	Yes	31 (25-35)	
	No	32 (27-38)	
**Income (US $)**			<.001
	<50,000	31 (25-37)	
	≥50,000	33 (30-40)	
**Parent education**			<.001
	High school or less	28 (24-32)	
	Any college or more	33 (30-39)	
**Parent language**			<.001
	English	32 (28-38)	
	Spanish	26 (18-31)	

a N/A: not applicable.

## Discussion

### Principal Findings

This exploratory descriptive study uniquely examined and highlighted the significant differences in technology-related characteristics between English- and Spanish-speaking parents among households with children with asthma. Spanish-speaking parents were less likely to report having high-speed internet and had higher concerns about paying for the internet during the pandemic, although these findings were not significant when adjusted for income status. In the adjusted models, Spanish-speaking parents remained more likely to report concern about data privacy when using technology for their child’s health. These findings are crucial in aiding the design and implementation of digital health care for pediatric patients and for prioritizing resources and the concern of parents to ensure the equitable use of these tools by families.

Although some technology differences might be related to economic status, they might be also associated with other important factors that shape families’ interests and capacity to use digital tools in asthma management [28-30]. The differences in digital connectivity in the household, internet affordability, perceptions of data privacy, and digital literacy have a potential influence on how families might engage with asthma digital technology. The findings emphasized the need for understanding which characteristics might be potential facilitators or barriers to using digital tools in pediatric asthma clinical care.

Knowing the household resources for digital connectivity was critical for understanding families’ access to digital health tools. Asthma predominantly affects those in low socioeconomic statuses, and digital health equity has become an increasing issue for those in historically marginalized communities as technology use has expanded [12,18]. Our results were similar to national trends that found nearly 90% of households possessed a smartphone, and there were no significant differences between households for smartphone and tablet ownership [31]. However, racial and ethnic disparities in the types of internet access, reported by the Pew Research Center, were also evident in the parent’s chosen language in our results. The national survey in 2021 found that Hispanic adults were less likely to have a home broadband connection and more likely to report smartphone-only internet [31]. Since most Spanish-speaking parents also identified as Hispanic or Latino/Latina in our study, we found similar patterns of fewer Spanish-speaking parents owning a smartphone data plan or high-speed internet service at home than English-speaking parents. The differences in digital connectivity by parent language could be related to affordability and income; although for parents with school-aged children in public schools, there was a program for no-cost internet [32]. Another barrier might also be the lack of high-speed broadband services in neighborhoods where these families live [33,34].

An additional factor shaping differing technology characteristics might be digital literacy. The “digital literacy” term has been interchangeably used with eHealth literacy but was broadly defined as “an individual’s ability to access, understand, and engage with digital healthcare materials or technology to contribute to quality of life.” [23,24] The eHEALS is the most widely used eHealth literacy measurement available in different languages and for different age groups [22,35].

Our findings showed significant associations with socioeconomic status and other social determinants of health, like race, income level, education level, and preferred parent language. Since eHealth, or digital literacy, has theoretical foundations in health literacy and self-efficacy, these findings were not surprising. The eHEALS scores mostly varied in the 30s within a potential range of 8 to 40. The differences in median eHEALS scores were largest by parent education level and preferred language in our study, but the aggregate eHEALS scores were similar to previous studies of older adults with chronic disease who had familiarity with health care or with using web-based resources [25,36,37]. While the variation of eHEALS scores was minimal in our study, other literature has emphasized the eHEALS’ use longitudinally to evaluate whether exposure to digital health interventions improves participants’ scores. Using eHEALS or other assessments of digital literacy might be a helpful way to prioritize resources for supporting patients’ use of technology in clinical care [38].

The findings’ implications for our clinical population are important. While having high-speed internet and concerns about paying for internet were no longer significant in the adjusted model controlling for household income, concerns about data privacy remained significant by language. Aside from access barriers to digital engagement, patients might have differing views on why they find using digital tools valuable in health care and their comfort with what, how, and why health information is shared. For example, in an SMS text message–based mental health intervention, researchers found that English speakers reported increased introspection with the intervention, but Spanish speakers highlighted feelings of social support [17]. While we did not evaluate motivations for engaging in digital health tools in this study, ensuring that our digital interventions align with why patients might engage with them is necessary for sustaining digital health approaches [39].

### Limitations

There are limitations to this study. This was a single health system study of patients recruited from pediatric pulmonary clinics and may not be generalizable to patients with asthma at other institutions or patients not managed by a specialist. Although eHEALS is the most used validated measure for digital literacy, the questions focus on a person’s familiarity with internet use and navigation, which may not be indicative of other skills around digital literacy that have evolved with the use of smartphones and mobile apps. Given the limited sample size, we also could not use a multivariable model to evaluate for confounding between parent language, income level, and parent education level in our study population.

### Conclusions

Our study found that the integration of digital tools into health care will potentially require adaptations to improve access to digital devices, resources for high-quality digital connectivity, and assistance for navigating digital tools for this patient population. Examining and comparing these factors to support the equitable use of digital tools in asthma care is necessary to ensure that our socioeconomically and language-diverse populations with asthma receive high-quality asthma care and support for self-management.

## Supplementary material

10.2196/48822 Multimedia Appendix 1 Characteristics of parent/caregiver and child by survey participation type (N=197).

10.2196/48822 Multimedia Appendix 2 Survey instrument.

## Abbreviations

aOR: adjusted odds ratio
eHEALS: eHealth Literacy Scale
*ICD-10*: *International Classification of Diseases, Tenth Revision*
