# Supplementary material for: Digital Technology Characteristics and Literacy Among Families With Children With Asthma: Cross-Sectional Study
Source: JMIR Pediatr Parent. 2023 Nov 29;6:e48822. doi: 10.2196/48822 (PMC10702171; doi:10.2196/48822)
Supplement: Multimedia Appendix 1 [file pediatrics-v6-e48822-s001.docx]

**Supplement Table 1. Characteristics of Parent/Caregiver and Child by Survey Participation Type (n=197)**

|  | Overall Sample, n (%)  n=197 | In-person  n (%)  n=81 | Online  n (%)  n=116 | p-value |
| --- | --- | --- | --- | --- |
| **Parent Age (Median, IQR)**  (n=181) | 37 (32, 43) | 35 (31, 42) | 41 (37, 45) | 0.56 |
| **Parent Gender^1^** (n=193) |  |  |  |  |
| Male | 20 (10.4%) | 9, (11.5%) | 11, (9.6%) | 0.65 |
| Female | 172 (89.1%) | 69, (88.5%) | 103, (89.6%) |  |
| **Parent Race** (n=191) |  |  |  |  |
| Other (Asian, American Indian/Alaskan  Native, Hawaiian/PI) | 49 (24.4%) | 25, (31.6%) | 24, (21.6%) | 0.14 |
| White | 67 (37.1%) | 22, (27.8%) | 45, (40.5%) |  |
| Black or African American | 75 (38.6%) | 33, (40.5%) | 42, (37.8%) |  |
| **Parent Ethnicity, Hispanic/Latino/a** (%Yes) | 93 (47.2%) | 41, (51.9%) | 52, (46.0%) | 0.53 |
| **Parent Education** |  |  |  |  |
| High School or Less | 82 (41.6%) | 36, (44.4%) | 46, (39.1%) | 0.46 |
| Any College or More | 115 (58.4%) | 45, (55.6%) | 70, (60.9%) |  |
| **Estimated Annual Household Income** (n=180) |  |  |  |  |
| < $50,000 | 129 (65.5%) | 54, (73.0%) | 74, (70.5%) | 0.72 |
| > $50,000 | 51 (25.9%) | 20, (27.0%) | 31, (29.5%) |  |
| **Child Gender** |  |  |  |  |
| Male | 124 (62.9%) | 42 (52.0%) | 82, (70.4%) | 0.01 |
| Female | 73 (37.1%) | 39 (48.0)% | 34, (29.6%) |  |
| **Child Race** (n=191) |  |  |  |  |
| Other (Asian, American Indian/Alaskan Native, Hawaiian/PI) | 37 (18.8%) | 17, (21.5%) | 20, (18.0%) | 0.61 |
| White | 75 (38.1%) | 28, (35.5%) | 47, (42.4%) |  |
| Black or African American | 79 (40.1%) | 35, (43%) | 44, (39.6%) |  |
| **Child Ethnicity, Hispanic/Latino/a** (%, yes) | 92 (46.7%) | 41, (53.2%) | 51, (43.6%) | 0.18 |
| **Child Grade** (2020-2021) (n=196) |  |  |  |  |
| Kindergarten-8^th^ grade | 174 (88.8%) | 71, (87.6%) | 103, (89.6%) | 0.67 |
| 9^th^-12^th^ grade | 22 (11.2%) | 10, (12.4%) | 12, (10.4%) |  |
| **Asthma Diagnosis** (n=196) |  |  |  |  |
| Mild | 27 (13.8%) | 11 (13.8%) | 16 (13.8%) | 0.2 |
| Moderate | 51 (26.0%) | 26 (32.5%) | 25 (21.6%) |  |
| Severe | 118 (60.2%) | 43 (53.8%) | 75 (64.7%) |  |

^1^ One participant selected “other/preferred not to answer.”
