# Supplementary material for: Digital Technology Characteristics and Literacy Among Families With Children With Asthma: Cross-Sectional Study
Source: JMIR Pediatr Parent. 2023 Nov 29;6:e48822. doi: 10.2196/48822 (PMC10702171; doi:10.2196/48822)
Supplement: Multimedia Appendix 2 [file pediatrics-v6-e48822-s002.docx]

# Pediatric Asthma and Digital Health Access during COVID-19 Pandemic Survey [1]

1. What is YOUR age? [18+]

2. What is YOUR gender?

- Male
- Female
- Other (how do you self-identify your gender? [free text])
- Prefer not to answer

3. Are YOU of Hispanic, Latino, or Spanish descent?

- Yes
- No

4. What is YOUR race? (Select all that apply)

- American Indian/Alaska Native
- Asian
- Black or African American
- Hawaiian/Pacific Islander
- White
- Prefer to self-describe (how do you self-describe YOUR race? [free text])

5. What is the highest grade or level of school you have completed?

- Less than high school (8^th^ grade or less)
- Some high school (9^th^-12^th^ grade, no diploma)
- High school graduate or GED completed
- Some college credit but no degree
- College degree

6. What is your estimated annual household income? This is the total earning over the last year.

- Less than $20,000
- $20,000-$34,999
- $35,000-$49,999
- $50,000-$74,999
- $75,000-$99,999
- More than $100,000
- Prefer not to answer

7. Is YOUR CHILD of Hispanic, Latino, or Spanish descent?

- Yes
- No

8. What is YOUR CHILD’S race? (Select all that apply)

- American Indian/Alaska Native
- Asian
- Black or African American
- Hawaiian/Pacific Islander
- White
- Prefer to self-describe (how would you self-describe your child’s race? [free text])

9. Please select the school grade level of your child was during 2020-2021 [none, daycare, preschool, kindergarten, 1-12]

10. Would you describe the health difficulties caused by [his/her/their] asthma as minor, moderate, or severe? [2]

- Minor
- Moderate
- Severe

11. Which of the following devices does your your family use at home? (Select all that apply [3])

- Desktop or laptop
- Smartphone
- Tablet or other portable wireless computer
- Some other type
- None of the above

12. Which type of activities does your child use the above devices for? (Select all that apply)

- Remote learning
- Entertainment (examples: YouTube, games)
- Communication with family/friends
- Other (what other types of activities does your child use the above devices for? [free text])
- 13. Which of the following types of internet access do you have at home? (Select all that apply [3]) Cell phone data plan for a smartphone or other mobile device
- High speed internet service (examples: cable, fiber optic, or DSL service)
- Satellite internet service
- Some other service (what other type of internet access do you have at home? [free text])

14. How much, if at all, did you worry about being able to pay for your internet connection at home during the CORONAVIRUS/COVID-19 pandemic?

- A lot
- Some
- Not too much
- Not at all
- Do not have to pay for internet

Please choose how much you agree with the following statements:

|  | Strongly Disagree | Disagree | Neutral | Agree | Strongly Agree |
| --- | --- | --- | --- | --- | --- |
| 15. I know how to find helpful health resources on the Internet |  |  |  |  |  |
| 16. I know how to use the Internet to answer my health questions |  |  |  |  |  |
| 17. I know what health resources are available on the Internet |  |  |  |  |  |
| 18. I know where to find helpful health resources on the Internet |  |  |  |  |  |
| 19. I know how to use the health information I find on the Internet to help me |  |  |  |  |  |
| 20. I have the skills I need to evaluate the health resources I find on the Internet |  |  |  |  |  |
| 21. I can tell high quality from low quality health resources on the Internet |  |  |  |  |  |
| 22. I feel confident in using information from the Internet to make health decisions |  |  |  |  |  |

23. How interested are you in using technology (example: video clinic visits, cell phone apps, sensors on inhaler to check if medicine taken, etc.) for managing your child’s asthma?

- A lot
- Some
- Not too much
- Not at all

24. How concerned are you about the privacy of your data when using technology for your child’s health?

- A lot
- Some
- Not too much
- Not at all

References:

1. National Survey of Children's Health 2019 [database on the Internet]. The Child and Adolescent Health Measurement Initiative. [cited 6/19/2021]. Available from: https://www.childhealthdata.org/learn-about-the-nsch/topics_questions/2019-nsch-guide-to-topics-and-questions.

2. National Survey of Children's Health 2003 [database on the Internet]. [cited 6/19/2020]. Available from: https://nschdata.org/browse/survey/results?q=573.

3. Lewis J. 2016 American Community Survey Content Test Evaluation Report: Computer and Internet Use. Washington, D.C.: U.S. Department of Commerce, Economics and Statistics Administration, U.S. Census Bureau, 2017 Contract No.: ACS17-RER-09.
